# Differences in the Natural Swimming Behavior of *Schizothorax prenanti* Individual and Schooling in Spatially Heterogeneous Turbulent Flows

**DOI:** 10.3390/ani13061025

**Published:** 2023-03-10

**Authors:** Minne Li, Min Chen, Weixiong Wu, Jia Li, Ruidong An

**Affiliations:** 1 State Key Laboratory of Hydraulics and Mountain River Engineering, College of Water Resource & Hydropower, Sichuan University, Chengdu 610065, China; 2 Guangxi Key Laboratory of Water Engineering Materials and Structures, Guangxi Water Conservancy Research Institute, Nanning 530023, China

**Keywords:** ascent paths, swimming behavior, open-channel flume, hydrodynamic preferences, husbandry environments

## Abstract

**Simple Summary:**

Exploring the behavioral strategies of fish in response to complex hydrodynamic environments is an important scientific focus in fish habitat enrichment research. The results of this research indicated that (1) the swimming speed of fish schooling (three fish) was significantly lower than that of individual fish, (2) fish schools performed obvious slow-speed exploration behavior during upstream migration, and (3) fish mainly tended to occupy low and medium flow velocity areas. The results of this research enrich the knowledge of fish behavioral responses to spatially heterogeneous turbulent flows, which is an important aspect for developing reliable and accurate estimates of fish passage facilities and husbandry environments.

**Abstract:**

Spatially heterogeneous turbulent flow refers to nonuniform flow with coexisting multiple flow velocities, which is widely distributed in fish natural or husbandry environments, and its hydraulic parameters affect fish swimming behavior. In this study, a complex hydrodynamic environment with three flow velocity regions (low, medium, and high) coexisting in an open-channel flume was designed to explore volitional swimming ability, the spatial-temporal distribution of fish swimming trajectories, and the range of preferred hydrodynamic parameters of *Schizothorax prenanti* individual and schooling (three fish). The results showed that the swimming speed of individual fish during upstream migration was significantly higher than that of fish schools (*p* < 0.05). The swimming trajectories of fish schooling showed that they spent more time synchronously exploring the flow environment during upstream migration compared with individual fish. By superimposing the fish swimming trajectories on the environmental flow field, the range of hydrodynamic environments preferred by fish in complex flow fields was quantified. This research provides a novel approach for investigating the natural swimming behavior of fish species, and a theoretical reference for the restoration of fish natural habitats or flow enrichment of husbandry environments.

## 1. Introduction

The construction of water conservancy and hydropower projects provides important functions such as flood control, power generation, and irrigation [1], while it also alters the ecological connectivity of natural rivers, blocks the upstream and downstream migratory routes required by fish in different life history stages [2], and further affects fish reproduction and gene exchange [3,4,5,6], resulting in a dramatic decline in migratory fish biodiversity and population density [7]. Fish passage facility, as an effective engineering measure for the restoration of fish habitat fragmentation [8,9,10,11,12], can mitigate the ecological–environmental impact of anthropogenic structures on river ecosystems to some extent [13,14]. However, the monitoring of the fish passing effect showed that the operation of most domestic fish passage facilities is not ideal [15]. Multiplication release is also an important method to protect damaged biological resources and maintain the sustainable development of fisheries [16]. However, due to the simple husbandry environments, cultured fish often suffer from low welfare levels [17], and mitigation efforts are hampered by poor knowledge of the response relationship between fish swimming behavior and the complex hydrodynamic environment [18,19,20].

Fish swimming behavior is affected by various external environmental factors and internal physical and chemical mechanisms, among which hydrodynamic motion is generally considered to be particularly important [4,21,22]. For example, juvenile Atlantic salmon smolts (*Salmo salar*) use the fine-scale flow velocity and turbulent kinetic energy within their sensory range as effective guidance information for downstream migration, and the flow direction has a key impact on fish swimming behavior [18]. Moreover, to maintain steady swimming, fish actively avoid turbulent flow areas with high vorticity and Reynolds shear stress [23], because when the ratio of the turbulence length scale to fish body length exceeds a certain upper limit, the fish will experience a loss of navigation stability (spill) [24]. Field studies have found that fish prefer to swim within a specific hydraulic range during their upstream or downstream spawning migration to save energy [25]. Furthermore, studies have shown that most of the current tests of fish swimming ability are evaluated by forced swimming in a closed and uniform flow environment [8,14,26]. Uniform flow is rarely present in the natural habitats of fish, and the closed test environment limits the volitional swimming performance of fish. As a result, those studies underestimated the real swimming ability of fish and led to the design of fishways with conservative flow velocities [27]. Compared to swimming alone, individual fish in schools can use the vortex trail shed by adjacent fish to reduce hydrodynamics resistance [28], so fish mainly adopt schooling patterns for migration in nature [29]. Therefore, exploring the behavioral responses of individual fish and schooling to various complex hydrodynamic environments is of great significance for deepening the understanding of fish behavior and promoting biodiversity protection [10,30].

*Schizothorax prenanti*, Tchang, 1930, was selected as the research object to investigate the swimming behavioral responses of individual fish and schooling to various complex hydrodynamic environments. *Schizothorax prenanti*, belonging to the *Cyprinidae* family and *Schizothoracids* subfamily, is mainly distributed in the Jinsha River and other tributaries in the upper reaches of the Yangtze River. It is an economically valuable species and primarily inhabits in junction of rapid and slow flows with high oxygen content [31]. Affected by cascade hydropower development, the aquatic ecological environment in the upper reaches of the Yangtze River is vulnerable, with rare and highly endemic fish species [32]. The lack of effective fish passage facilities has led to a rapid decline in the population of *S. prenanti*, and now they are on the verge of extinction [21]. There have been few scientific and quantitative studies of the volitional swimming ability and preferred hydrodynamic range of *S. prenanti* individual and schooling in Southwest China [33,34].

In this study, by setting up the complex flow field characteristics with different flow velocity regions in an open-channel flume, the differences in the natural swimming behavior of *S. prenanti* individual and schooling were studied, including volitional swimming ability, spatial-temporal distribution of swimming trajectories and range of preferred hydrodynamic parameters. The main objective of the present study was to investigate swimming behavioral responses of individual and schooling of *S. prenanti* to various complex hydrodynamic environments. This research not only enriches the understanding of the behavioral responses of *S. prenanti* to spatially heterogeneous turbulent flows but also provides a novel research method for studying the natural swimming behavior of other fish species.

## 2. Materials and Methods

### 2.1. Experimental Apparatus

To investigate the volitional swimming behavior of individual fish and schooling during upstream migration in this study, an open-channel flume with spatially heterogeneous turbulent flow was designed. The functions and geometric dimensions of each part of the flume have been described in detail by Li et al. (2022) [33].

**Figure 1:**
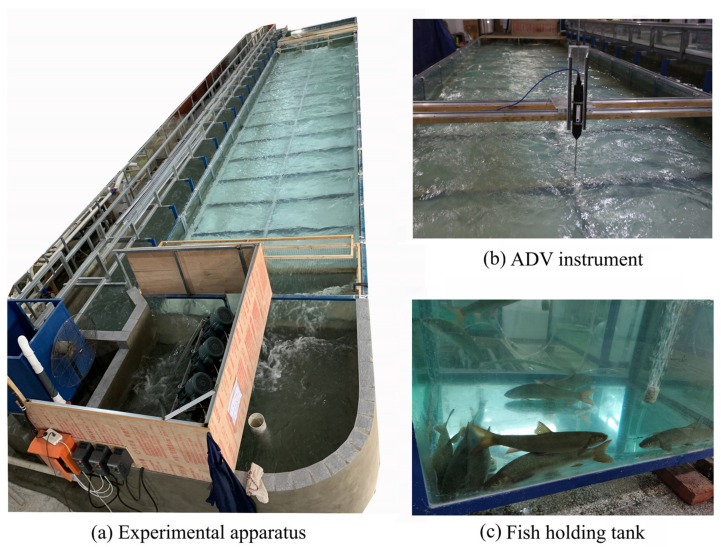
Real device: (**a**) experimental apparatus in the State Key Laboratory of Hydraulics and Mountain River Engineering of Sichuan University, (**b**) Acoustic Doppler Velocimeter (ADV) instrument, and (**c**) *Schizothorax prenanti* holding tank.

The flow circulation power in the flume was maintained by three electric propellers installed downstream (Figure 1a). Pushed by these propellers, a constant flow rate of approximately 0.60 m^3^/s flowed from the side concrete channel and entered the head tank through the discharge intake. The turbulent flow at the head tank was rectified by the flow straightener, and then entered the three inlets with different numbers of rack bars to form different flow velocity regions. Specifically, the numbers of rack bars at inlets 1, 2, and 3 were 7, 5, and 0, respectively, thus forming low, medium, and high flow regions, respectively (Figure 1a).

### 2.2. Hydraulic Characteristics

**Figure 2:**
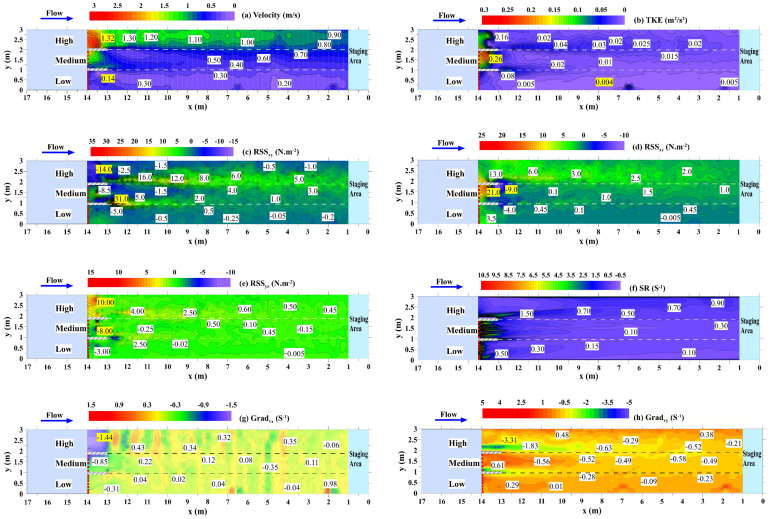
Contour plots of different hydraulic parameters: (**a**) flow velocity (m/s), with the white dots indicating the ADV measurement points and the white dashed lines indicating the flow regime boundaries; (**b**) turbulent kinetic energy (TKE, m^2^·s^−2^); Reynolds shear stress in three planes: (**c**) RSS_xy_, (**d**) RSS_xz_, and (**e**) RSS_yz_, N**^.^**m^−2^; (**f**) strain rate (SR, s^−1^); and (**g**) velocity gradients along (Grad_vx_, s^−1^) and (**h**) perpendicular to the direction of water flow (Grad_vy_, s^−1^).

The three-dimensional hydraulic parameters in the swim test area, such as flow velocity, turbulent kinetic energy, and Reynolds shear stress, were measured by the Acoustic Doppler Velocimeter instrument (ADV, Figure 1b). The measurement frequency of each point was 50 Hz, and the measurement time was 60 s [35]. The velocity information was only measured at 10 cm above the bottom of the flume, since visual observation through the glass sidewall revealed that the fish mainly swam in this horizontal plane. The measurement intervals along and perpendicular to the flow direction are 0.25 m and 0.10 m, respectively, with a total of 1421 measurement points (white dots in Figure 2a). After the measurement, the raw ADV data were post-processed and filtered using the Win ADV program [36]. The calculation formulas of turbulent kinetic energy (k), Reynolds shear stress ($\tau_{uv}^{}$), and flow velocity gradient ($Grad_{vi}$) were as follows.

(1) $$k=(\bar{u{{}^{\prime }}^{2}}+\bar{v{{}^{\prime }}^{2}}+\bar{w{{}^{\prime }}^{2}})/2$$

The focal flow velocity was expressed as the sum of the time-averaged ($\bar{u}$, $\bar{v}$, $\bar{w}$) and fluctuating ($u^{\prime }$, $v^{\prime }$, $w^{\prime }$) components.

(2) $$\tau_{uv}^{}=-\rho \bar{u^{\prime }v^{\prime }}$$

RSS appears when two water masses or layers with different velocities are adjacent and can be partitioned into three planes: horizontal (*xy* plane), vertical (*xz* plane), and transverse (*yz* plane).

(3) $$Grad_{vi}=\Delta v_{i}/\Delta s_{i}$$

where $\Delta V_{i}$ is the flow velocity difference along the flow direction and $\Delta S_{i}$ is the corresponding distance difference.

Computational Fluid Dynamics software Flow-3D (Supplementary File S1) was used to obtain the strain rate of turbulent flow [37] and was defined as:

(4) $$SR_{ij}=(\partial u_{i}/\partial x_{j}+\partial u_{j}/\partial x_{i})/2$$

where $u_{i}$ and $u_{j}$, respectively, denote the velocities in different directions.

### 2.3. Fish Species Husbandry

In this study, cultured *S. prenanti* in the reproductive period was selected as the research object, and the experimental fish samples (*n* = 60) were provided by the Fisheries Institute of Sichuan Province. Healthy fish were placed in oxygenated water bags and then transported to the State Key Laboratory of Hydraulics and Mountain River Engineering of Sichuan University for temporary holding. After a week of adaptation, the physiological state of *S. prenanti* was stabilized for testing. The temporary holding tank (Figure 1c) was a square glass tank with dimensions of 1.5 m × 1.5 m × 1.5 m (length × width × height). During the holding period, an air pump continuously supplied oxygen to the tank. The dissolved oxygen was maintained at 6.5–8.5 mg/L, and 10% of the total water volume was replaced every day to maintain the quality of the water environment (7 < pH < 8). The water temperature was maintained at approximately 18.83 ± 0.24 °C (mean ± SD) by using medical-grade constant-temperature ice packs. After the test, the range of the body length and weight of each fish were measured. The body length [*BL*] range was 26.9 ± 2.4 cm (mean ± SD), the fork length [*FL*] was 29.6 ± 2.5 cm, the total length [*TL*] was 32.3 ± 2.7 cm, and the body weight range was 304.2 ± 77.2 g.

### 2.4. Experimental Methods

The minimum value of the flow velocity in the flume should be higher than the fish induced flow velocity to generate rheotaxis behavior, and the maximum value should be less than the fish burst swimming speed to prevent the formation of a flow velocity barrier. Fu et al. (2013) [38] tested the swimming ability of *S. prenanti* with a body length of 0.29 ± 0.01 m in a closed swimming chamber with a water temperature of 19.40 °C, and found that the ranges of induced, critical, and burst swimming speeds were 0.01–0.13 m/s, 0.65–1.09 m/s, and 0.85–1.53 m/s, respectively. Since the fish body length and water temperature in the test environment were similar to those in our study, the flow velocities in low, medium, and high flow regions in the swim test area were, respectively, set in the ranges of induced, critical and burst swimming speeds of *S. prenanti* based on the results of Fu et al. [38].

Before the experiment, healthy fish were randomly selected from the holding tank and placed into the staging area for adaptation to eliminate the stress response. After half an hour of acclimatization, the removeable screen was raised, and the experimental fish entered the swim test area to swim volitionally. The complete behavioral process of the experimental fish was recorded by four high-speed video cameras installed above the flume [33]. When the experimental fish passed the swim test area and entered any of the three upstream inlets, the test was considered completed, and the duration of the experiment was one hour. Passage time was defined as the time from when a fish entered the test area to when it entered the upstream inlets, and the passage time of each fish (both individual and schooling fish experiments) was recorded independently. In tests of individual fish, one fish was released at a time, and the test was performed for 60 different fish. After the individual fish experiment, the experimental fish were kept for one week to recover before the schooling experiment. In the fish schooling test, three fish were released at a time, and the test was repeated 20 times with 60 different fish.

### 2.5. Swimming Performance

#### 2.5.1. Swimming Capability

In this study, the difference in swimming speed between individual fish and schooling was compared and analyzed. The swimming speed [39] of individual and schooling fish (relative to flowing water) is defined as:

(5) $$\vec{V_{fish}}=\vec{V_{ground}}-\vec{V_{water}}$$

where $\vec{V_{water}}$ (m/s) is the average water velocity against which the fish swims, $\vec{V_{ground}}$ (m/s) indicates the speed of the fish relative to the geodetic reference system, and the calculation formula of $\vec{V_{ground}}$ is as follows:

(6) $$\vec{V_{ground}}=\sqrt{{\left(x_{t}-x_{t-1}\right)}^{2}+{\left(y_{t}-y_{t-1}\right)}^{2}}/\Delta t$$

where $x_{t}$ and $y_{t}$ represent the coordinate position of the fish at time *t*, and Δt (1/30 s) is the time interval (s) between consecutive coordinate positions.

#### 2.5.2. Swimming Trajectory

The behavioral analysis software Logger Pro 3.16 was used to process the experimental video frame by frame (30 fps), by recording the two-dimensional coordinate position of the fish in each frame, and finally connecting the positions in time to obtain the complete fish swimming trajectory. In addition, a heatmap was used to display the spatial-temporal distribution characteristics of the swimming trajectories of individual fish and schooling. The cell size of the heatmap was set based on the average ground speed of fish (0.27 m/s) and the body length range of the test fish. Finally, the cell size in the heatmap was set to 0.25 m.

#### 2.5.3. Preferred Hydrodynamic Range

In this study, the trajectories of individual fish and schooling were coupled with the hydrodynamic environment to obtain the preferred hydrodynamic range during upstream migration. The first step in the specific statistical method was to divide the environmental hydraulic values into equal intervals (environmental flow), and then use the same interval to construct the probability density functions (PDFs) of fish occupancy hydraulics (selective flow). To analyze the preference and avoidance behavior of fish for different hydraulic values, we calculated the difference between the PDFs of selective and environmental flows in different intervals. When the difference was greater than zero, the case was regarded as a preference, whereas a value less than zero indicated avoidance, and a value equal to zero was considered no choice [40].

### 2.6. Data Analysis

The Spearman rank coefficient was used to evaluate the correlations between the fish transit time in each cell and the hydraulic variables. All statistical analyses were performed using IBM SPSS Statistics 22 software, and all tests were two-sided with a significance level of 0.05.

## 3. Results

### 3.1. Flume Hydraulics and Tolerances

The ranges of hydraulic parameters in each flow velocity region, including flow velocity (V, m/s), turbulent kinetic energy (TKE,m^2^·s^−2^), and Reynolds shear stress in three planes, (RSS_xy_, RSS_xz_, RSS_yz_, N**^.^**m^−2^), strain rate (SR, s^−1^), velocity gradient along (Grad_vx_, s^−1^), and perpendicular to the direction of water flow (Grad_vy_, s^−1^), are shown in Table 1. The contour plots of different hydraulic parameters are shown in Figure 2.

The flow velocity in the research area varied from 0.14 to 1.32 m/s; similar to the flow velocity, the spatial distribution and magnitude of the TKE differed in the three flow regimes; the RSS values in the three planes increased with increasing flow discharge, and the SR values in the main research area ranged from 0.07 to 6.50 s^−1^ [33]. For the velocity gradient along the flow direction (Grad_vx_) in Figure 2g, the ranges corresponding to the low, medium and high flow regions were −0.31–0.98 s^−1^, −0.85–0.22 s^−1^ and −1.44–0.43 s^−1^, respectively. Compared with Grad_vx_, the velocity gradient perpendicular to the flow direction (Grad_vy_) displayed a higher variation in amplitude (Figure 2h). The ranges corresponding to the low, medium and high flow regions were −0.28–0.29 s^−1^, −0.58–0.61 s^−1^, and −3.31–0.38 s^−1^, respectively.

### 3.2. Swimming Speed Difference

**Figure 3:**
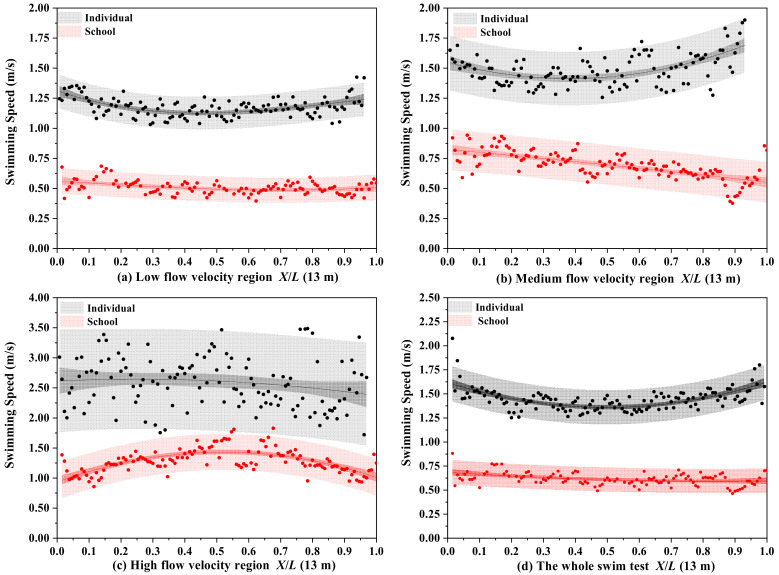
Swimming speeds with 95% confidence intervals (quantified by internal estimation) for individual *S. prenanti* and schooling in different flow velocity regions (low (**a**), medium (**b**), high (**c**) and the whole swim test area (**d**)). *x* is the position of the fish along the flow direction, and *L* (13 m) is the length of the swim test area in the open-channel flume.

The swimming speeds of individual fish and schools in different flow velocity regions (low, medium, and high) are shown in Figure 3.

In the low flow velocity region (Figure 3a), the swimming speed range of the individual fish was 1.18 ± 0.08 m/s (mean ± SD), and that of fish school was 0.51 ± 0.06 m/s (mean ± SD); in the medium flow velocity region (Figure 3b), the swimming speed range of the individual fish was 1.49 ± 0.13 m/s (mean ± SD), and that of the fish school was 0.68 ± 0.14 m/s (mean ± SD); in the high flow velocity region (Figure 3c), the swimming speed range of the individual fish was 2.63 ± 0.37 m/s (mean ± SD), and that of the fish schooling was 1.27 ± 0.23 m/s (mean ± SD). In combining all flow velocity regions (Figure 3d), it was found that the swimming speed range of individual was 1.45 ± 0.14 m/s (mean ± SD), and that of fish schooling was 0.62 ± 0.07 m/s (mean ± SD). According to the independent sample *t* test, the swimming speeds of the two experimental groups satisfied the condition of homogeneity of variance, and the mean swimming speed of individual fish was significantly faster than that of schooling fish in all flow velocity regions (*p* < 0.05).

### 3.3. Spatial-Temporal Distributions of Swimming Trajectories

#### 3.3.1. Swimming Trajectories

##### Fish Individual

**Figure 4:**
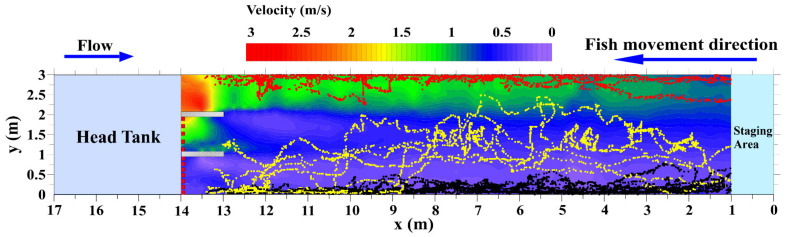
Superimposed diagram of typical swimming trajectories of individual *S. prenanti* on the contour line of flow velocity [33].

The classification and characteristics of individual fish swimming trajectories in Figure 4 were described in detail by Li et al. (2022) [33]. The volitional swimming behavior of 60 sample fish was investigated in the open-channel flume; 52 fish successfully moved into the test area, while 8 never left the staging area. For the routes (*n* = 52) recorded in the swim test area, the proportions of routes a, b, and c were 32.69%, 28.85%, and 13.46%, respectively, with other routes accounting for 25.00%.

##### Fish Schooling

**Figure 5:**
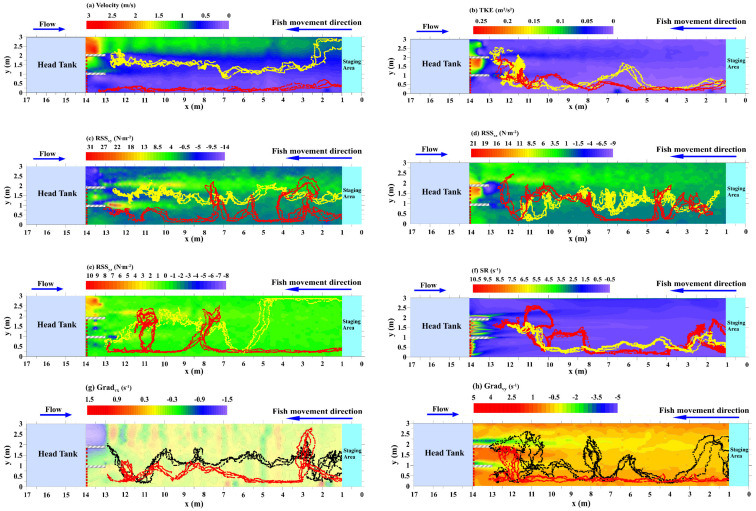
Diagrams of the typical swimming trajectories of *S. prenanti* schools superimposed on the distributions of different hydrodynamic parameters: (**a**) flow velocity (m/s); (**b**) turbulent kinetic energy (TKE, m^2^·s^−2^); Reynolds shear stress in three planes: (**c**) RSS_xy_, (**d**) RSS_xz_, and (**e**) RSS_yz_, N**^.^**m^−2^; (**f**) strain rate (SR, s^−1^); and (**g**) velocity gradient along (Grad_vx_, s^−1^) and (**h**) perpendicular to the direction of water flow (Grad_vy_, s^−1^).

The coupling results of the typical swimming trajectories of fish schools with different hydrodynamic parameters were shown in Figure 5, in which three trajectories with the same color represent that three fish were released together in one fish school trial. To provide a more comprehensive display of the characteristics of the trajectories of fish school, Figure 5 showed two independent groups of fish school trials with different hydrodynamic parameters; there were 2 × 8 = 16 groups of swimming trajectories in total. Figure 5 showed that fish school performed obvious “synchrony” and “coordination” exploration behaviors to the flow environment during the process of upstream migration. Compared with individual fish, fish schooling rarely swam in high-velocity areas and mainly preferred to swim in the low and medium velocity regions.

#### 3.3.2. Occupied Positions

**Figure 6:**
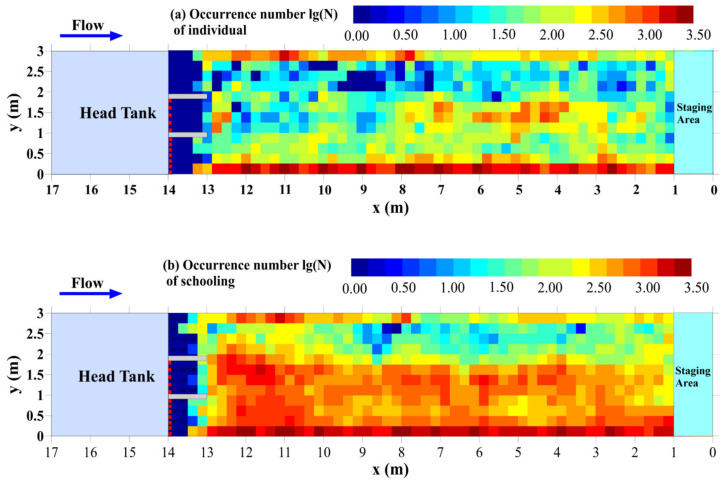
Heatmaps of individual *S. prenanti* and schooling in each cell $\left(0.25\times 0.25\;m\right)$ of the swim test area in the open-channel flume. (**a**) occurrence number lg(N) of individual and (**b**) Occurrence number lg(N) of schooling.

The total number (N) of occurrences of individual fish and schooling in each cell of the swim test area was shown in Figure 6. Individual fish and schools mainly preferred the low and medium flow velocity regions during upstream migration, and avoided the high flow velocity region. Both individuals and schools frequently appeared along the low flow velocity sidewall during upstream migration.

#### 3.3.3. Residence Time

**Figure 7:**
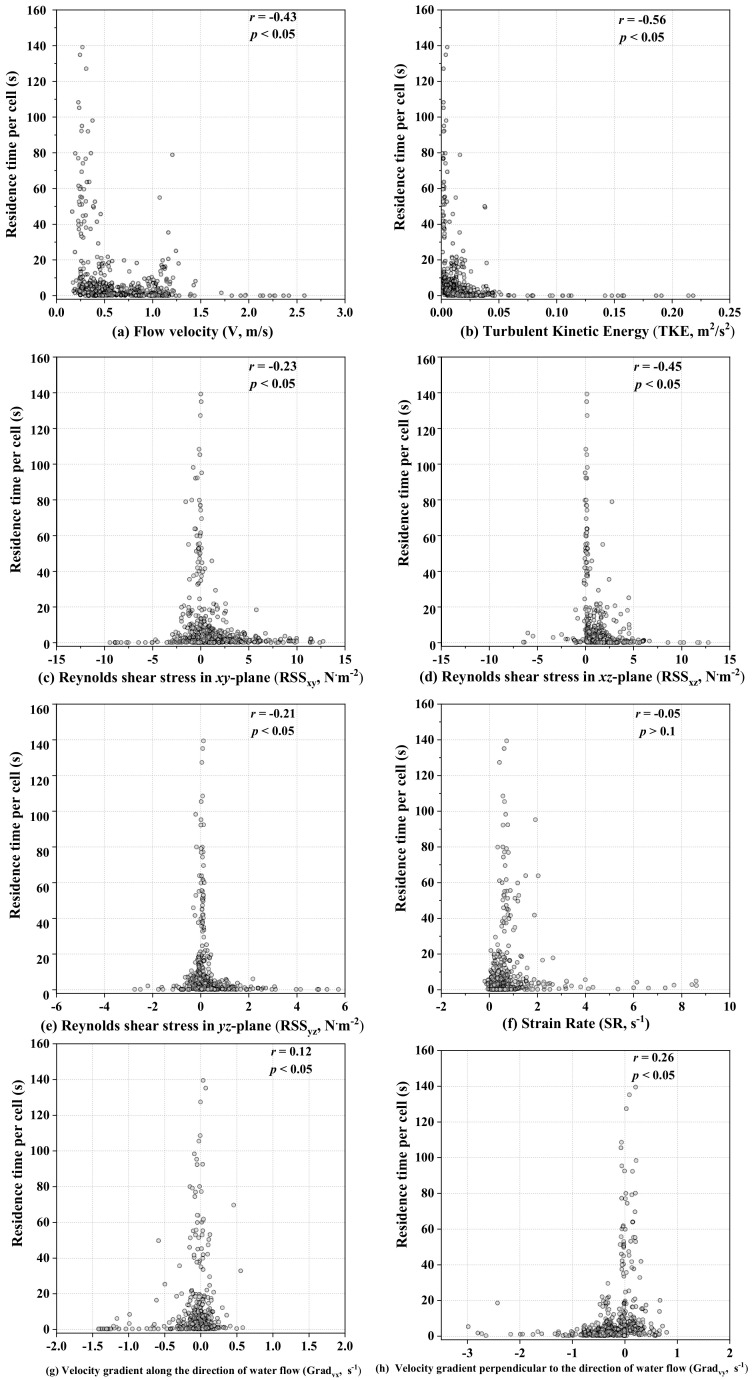
The relationship between the residence time of individual *S. prenanti* and the different hydrodynamic parameters: (**a**) flow velocity (m/s); (**b**) turbulent kinetic energy (TKE, m^2^·s^−2^); Reynolds shear stress in three planes: (**c**) RSS_xy_, (**d**) RSS_xz_, and (**e**) RSS_yz_, N**^.^**m^−2^; (**f**) strain rate (SR, s^−1^); and (**g**) velocity gradient along (Grad_vx_, s^−1^) and (**h**) perpendicular to the direction of water flow (Grad_vy_, s^−1^). *r* is the Spearman coefficient, and the *p* value represents the significance level.

**Figure 8:**
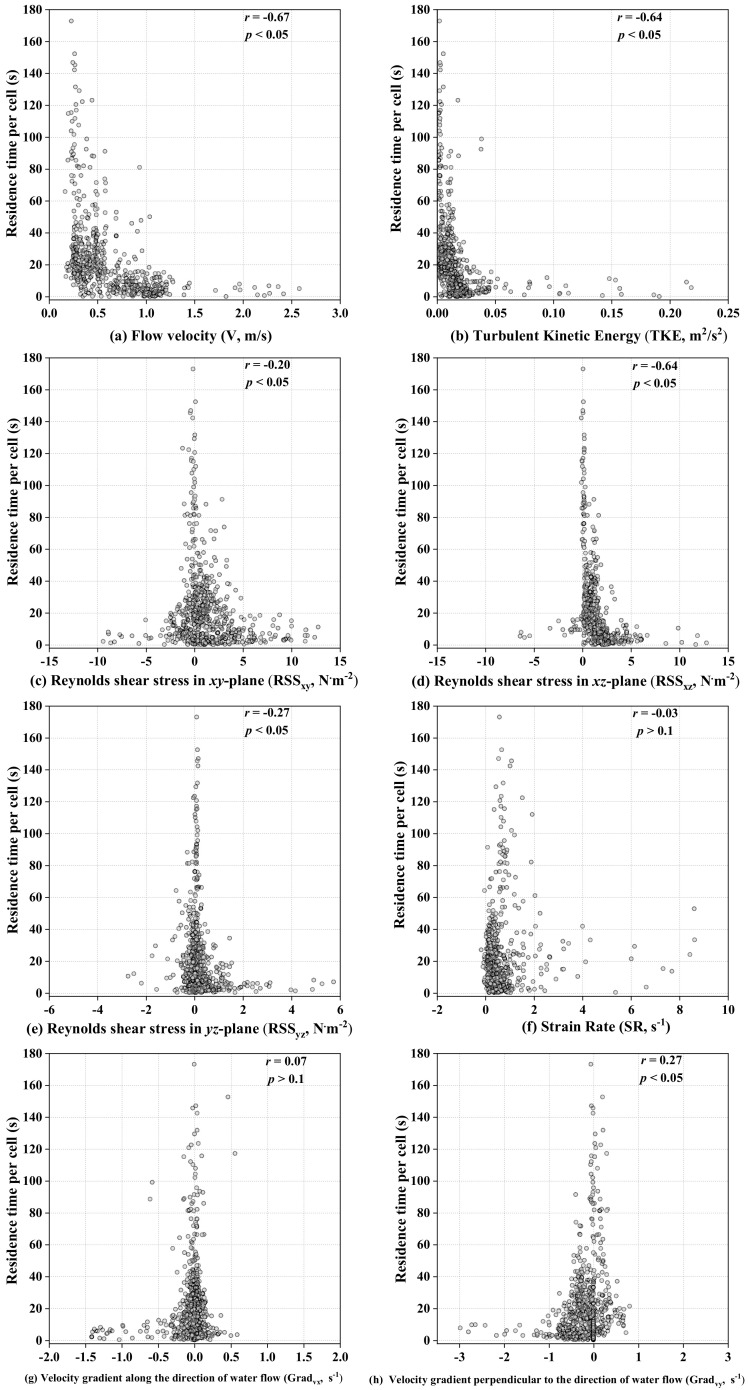
The relationship between the residence time of *S. prenanti* schools and the different hydrodynamic parameters: (**a**) flow velocity (m/s); (**b**) turbulent kinetic energy (TKE, m^2^·s^−2^); Reynolds shear stress in three planes: (**c**) RSS_xy_, (**d**) RSS_xz_, and (**e**) RSS_yz_, N**^.^**m^−2^; (**f**) strain rate (SR, s^−1^); and (**g**) velocity gradient along (Grad_vx_, s^−1^) and (**h**) perpendicular to the direction of water flow (Grad_vy_, s^−1^). *r* is the Spearman coefficient, and the *p* value represents the significance level.

Figure 7 showed the residence time of individual fish in different cells and the corresponding hydrodynamic ranges, and the influence of different hydraulic parameters on the residence time of individual fish was explained in detail by Li et al. (2022) [33]. Figure 8 showed the residence time of fish school and the corresponding hydrodynamic ranges in different cells. Compared with that for individual fish, the flow velocity occupied by fish school was wider and more discrete; it was approximately distributed in the range of 0.25–0.55 m/s, and there was a significant negative correlation between residence time and flow velocity (r = −0.67, *p* < 0.05). The occupied range of turbulent kinetic energy (<0.05 m^2^·s^−2^) was similar to that of the individual fish, and there was a significant negative correlation with the residence time (r = −0.64, *p* < 0.05). The occupied ranges of Reynolds shear stress on the three planes (*xy*, *xz*, and *yz*) were similar to those for individual fish, which were −3–5 N·m^−2^, 0–5 N·m^−2^ and −1–2 N·m^−2^, respectively, and there was a significant negative correlation between the Reynolds shear stress in the three planes and the residence time (*p* < 0.05).

As was the case for the individual fish, the occupied strain rate range of fish school was approximately 0–2 s^−1^, and there was no significant correlation with the residence time (*p* > 0.1). The velocity gradients along and perpendicular to the flow direction were positively correlated with the residence time, but the relationship with the residence time was not significant (*p* > 0.1).

#### 3.3.4. Spatial Distribution Probability and Passage Time

**Figure 9:**
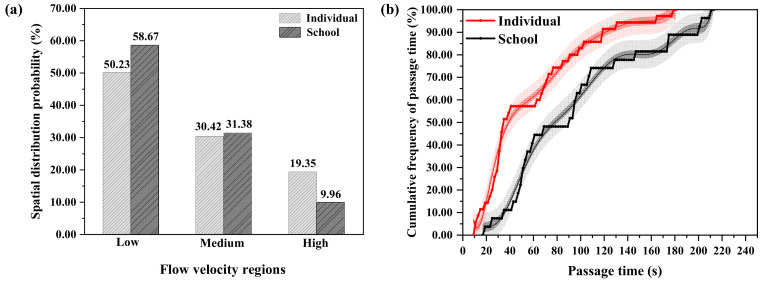
Spatial distribution probability and passage time: (**a**) Spatial distribution probability of *S. prenanti* individual and schooling in different flow velocity regions; (**b**) cumulative frequency of the passage time of *S. prenanti* individuals and schooling.

The percentages of the distributions of individual fish and schools in different flow velocity regions were calculated (Figure 9a). Individual fish and schools preferred low and medium velocity areas, and the probability of fish schools being distributed in high velocity region is less than that for individual fish. As shown in Figure 9b, the passage time range of fish schools was 94.43 ± 57.13 s (mean ± SD), and that for individual fish was 58.51 ± 43.96 s (mean ± SD). The result of an independent sample *t*-test showed that under the condition of homogeneity of variance (*p* > 0.05), the passage time of fish schools was significantly longer than that of individual fish.

### 3.4. Hydrodynamic Parameter Preferences

**Figure 10:**
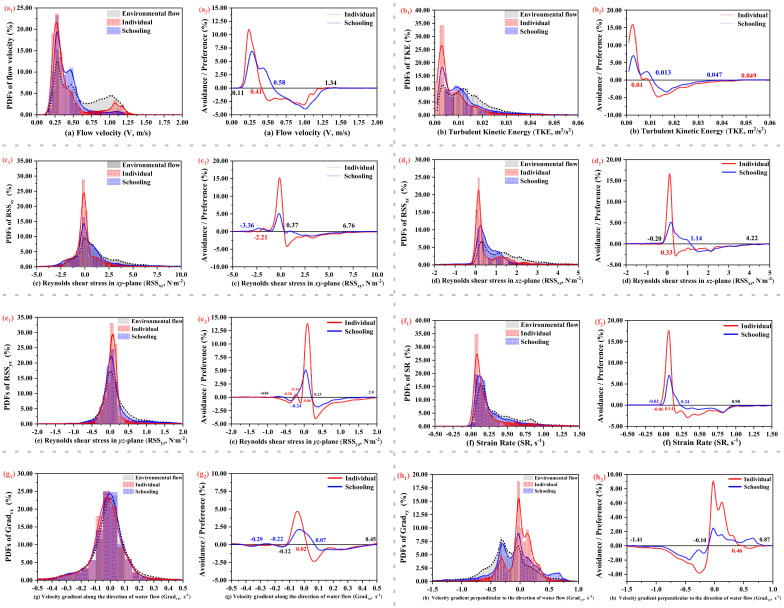
The preference and avoidance behaviors of fish to different hydraulic parameters during upstream migration: (**a**) flow velocity (m/s); (**b**) turbulent kinetic energy (TKE, m^2^·s^−2^); Reynolds shear stress in three planes: (**c**) RSS_xy_, (**d**) RSS_xz_, and (**e**) RSS_yz_, N**^.^**m^−2^; (**f**) strain rate (SR, s^−1^); and (**g**) velocity gradient along (Grad_vx_, s^−1^) and (**h**) perpendicular to the direction of water flow (Grad_vy_, s^−1^).

Figure 10 showed the ranges of different hydraulic parameters preferred by individual fish and schools during their upstream migration. Figure 10(a_1_) showed the distribution of the flow velocity in the environment (gray histogram) and the velocity distributions based on the preferences of individual (red histogram) and schools (blue histogram). As shown in Figure 10(a_2_), according to the calculated difference between selective and environmental flow velocities, the preferred flow velocity range (greater than zero) of fish individuals was 0.11–0.41 m/s, and the avoided flow velocity range (less than zero) was 0.41–1.34 m/s. For fish schools, the preferred flow velocity range was 0.11–0.58 m/s, and the avoided flow velocity range was 0.58–1.34 m/s. Finally, the flow velocity range preferred by fish was obtained based on the intersection of the preferences of individual fish and schools, which was 0.11–0.41 m/s. Similarly, the turbulent kinetic energy preferred by fish was 0–0.01m^2^·s^−2^ (Figure 10b), and the Reynolds shear stresses on the three planes (*xy*, *xz*, and *yz*) were −2.21–0.37 N·m^−2^ (Figure 10c), −0.20–0.33 N·m^−2^ (Figure 10d) and −0.06–0.23 N·m^−2^ (Figure 10e), respectively; the strain rate range was −0.16–0.14 s^−1^ (Figure 10f); and the velocity gradients along and perpendicular to the flow direction were −0.12–0.02 s^−1^ (Figure 10g) and −0.10–0.46 s^−1^ (Figure 10h), respectively.

## 4. Discussion

In this study, the swimming behavior of *S. prenanti* individual and schools was investigated in a spatially heterogeneous turbulent flow. By superimposing the fish swimming trajectories on the environmental flow field, the fish swimming speeds in different flow velocity regions were quantified, and the differences in the natural swimming behavior of *S. prenanti* individual and schools were compared and analyzed. In addition, by computing the differences between the PDFs of selective and environmental flows in different intervals, the preference and avoidance behaviors of fish to the range of hydrodynamic parameters were further revealed.

Fish schooling refers to individual fish gathering together to cooperate with each other to resist external environmental pressures, such as predation or defense against enemies, and the movement decisions of individual fish are often affected by nearby fish or other members of the school [41,42]. In this study, the swimming speeds of individual fish and schools in complex hydrodynamic environments were quantified and compared. The results showed that the swimming speed range of individual fish was 1.45 ± 0.14 m/s (mean ± SD) during upstream migration, and that of fish schools was 0.62 ± 0.07 m/s (mean ± SD). According to an independent sample *t* test, the swimming speed of the individual fish was significantly faster than that of the schooling (*p* < 0.05). Compared with individual fish, schooling fish displayed more “exploration behavior” in the environment when they moved together. Therefore, the passage time of fish schooling (94.43 ± 57.13 s) was significantly longer than that of individuals (58.51 ± 43.96 s). The reason for this difference may be that the swimming strategy, direction, time and other information associated with fish schools may result from “democratic” public decision-making, which synchronously balances the demand characteristics of different individual fish in schools. As a result, the swimming speed of fish schools is less than that of individual fish when moving alone, and their endurance increased two to six times when swimming in schools [28].

By designing a proper flow field with habitat environmental enrichment, the anxiety or abnormal behaviors of aquatic organisms in husbandry environments can be reduced, and environmental adaptability and the survival rate can be improved [17]. Fish mainly perceive hydraulic characteristics through their lateral line system and then perform behavioral responses accordingly [43]. Previous studies have shown that fish select their preferred hydrodynamic environment during volitional movement to reduce energy consumption [9,24,40]. In this study, the trajectories of individual fish and schools were superimposed on the environmental flow field to obtain the ranges of hydrodynamic preference and avoidance during upstream migration. The results showed that the preferred flow velocity range of fish was 0.11–0.41 m/s, and the avoided range was approximately 0.41–1.34 m/s. For spatial distribution probability, individual fish and schools mainly occupied low and medium flow velocity regions to maintain steady swimming. Fish preferred to swim in areas with low turbulent kinetic energy (approximately < 0.01m^2^·s^−2^) in our study. This result is consistent with that of Silva et al. (2020) [18], who found that when the turbulent kinetic energy was less than 0.03m^2^·s^−2^, it was suitable for Atlantic salmon navigation and stabilization; however, when the turbulent kinetic energy ranges from 0.03 to 0.24 m^2^·s^−2^, it will significantly increase the cost of locomotion of fish.

A relevant study has shown that there is a significant correlation between the Reynolds shear stress and the transit time of fish in vertical slot fishway [35]. In this study, the preferred Reynolds shear stress ranges of fish on the *xy*, *xz*, and *yz* planes were, respectively, −2.21–0.37 N·m^−2^, −0.20–0.33 N·m^−2^ and −0.06–0.23 N·m^−2^, and there was a significant negative correlation between the residence time of both individual fish and schools and the Reynolds shear stress on the three planes (*p* < 0.05). The correlation between the residence time and Reynolds shear stress on the *xz* plane was the highest among those in the three dimensions, suggesting that this variable has a strong influence on the behavioral response of fish to turbulent flow. For the strain rate, there was no significant correlation with the residence time of both individual fish and schools, and the preferred range was −0.16–0.14 s^−1^. In the absence of visual reference information, the flow velocity gradient was identified as the main evidence for fish to generate rheotaxis behavior and adjust swimming strategy to compensate for the displacement caused by the surrounding fluid [44]. This study showed that for the flow velocity gradient along the flow direction, the selection probability distributions of individual fish and schools were almost the same as the distribution of the flow velocity gradient, with no obvious preference behavior. However, for the velocity gradient perpendicular to the flow direction, individual fish and schools displayed obvious selection behavior, and the preferred range was −0.10–0.46 s^−1^. The reason for this difference may be that the change in velocity in this study mainly exists in the direction perpendicular to the flow, and the change in velocity along the flow direction was small.

This study investigated the differences in the volitional swimming behaviors of *S. prenanti* individual and schools in spatially heterogeneous turbulent open-channel flows, focusing on volitional swimming ability, spatial-temporal distribution of swimming trajectories, and range of preferred hydrodynamic parameters. The results provide a theoretical reference for effective flow field design in fish passage facilities and fish husbandry environments. Specifically, suitable dynamic flow husbandry environments can be designed to improve the physical and mental health of fish or the survival rate of multiplication release in the wild. While only specific turbulent flow characteristics, such as flow scale, orientation, intensity and periodicity, affect fish behavior, it is necessary to scientifically quantify the hydrodynamic thresholds that affect normal fish behavior by creating more adjustable factors in experimental flow environments. Furthermore, there are many internal and external factors that affect fish behavior, such as sound, light, substrate, water quality, and the physiological state of the fish itself. Therefore, to better create a suitable husbandry environment for fish growth and reproduction, comprehensive research should be conducted based on laboratory mechanisms and field experiments on various fish species in future work.

## 5. Conclusions

The results showed that the swimming speed range of individual fish was 1.45 ± 0.14 m/s (mean ± SD) and the range for fish schools was 0.62 ± 0.07 m/s (mean ± SD) during upstream migration. Compared with those of individual fish, the swimming trajectories of fish schools showed more “exploration behavior” in the environment when they moved together. Therefore, the passage time of fish schools (94.43 ± 57.13 s) was significantly longer than that of individual fish (58.51 ± 43.96 s). Furthermore, by superimposing the trajectories of individual fish and schools on the environmental flow field, it was found that the flow velocity range preferred by fish was 0.11–0.41 m/s; the preferred turbulent kinetic energy was 0–0.01m^2^·s^−2^; and the preferred Reynolds shear stresses on the *xy*, *xz*, *yz* planes were, respectively, −2.21–0.37 N·m^−2^, −0.20–0.33 N·m^−2^, and −0.06–0.23 N·m^−2^; the preferred strain rate was −0.16–0.14 s^−1^**;** and the preferred velocity gradients along and perpendicular to the flow direction were, respectively, −0.12–0.02 s^−1^ and −0.10–0.46 s^−1^. In summary, fish prefer to occupy areas with low flow velocities and low turbulence during the volitional movement. To improve the efficiency of fish passage and enhance the husbandry environment, a suitable flow environment should be created based on the preferred hydraulic ranges of the target species.

## Figures and Tables

**Table 1:** The ranges of hydrodynamic parameters (flow velocity (V, m/s), turbulent kinetic energy (TKE, m^2^·s^−2^), Reynolds shear stress in three planes (RSS_xy_, RSS_xz_, and RSS_yz_, N**^.^**m^−2^), strain rate (SR, s^−1^), and velocity gradients along (Grad_vx_, s^−1^) and perpendicular to the direction of water flow (Grad_vy_, s^−1^) corresponding to different flow velocity regions.

Flow Region	Velocity (m/s)	TKE (m^2^·s^−2^)	RSSxy	RSSxz	RSSyz	SR	Gradvx	Gradvy
			(N·m^−2^)	(N·m^−2^)	(N·m^−2^)	(s^−1^)	(s^−1^)	(s^−1^)
Low	0.14–0.38	0.004–0.08	−5.00–0.50	−4.35–3.50	−3.00–2.75	0.07–8.00	−0.31–0.98	−0.28–0.29
Medium	0.33–0.78	0.01–0.26	−8.50–31.00	−9.00–21.00	−8.00–4.80	0.08–8.50	−0.85–0.22	−0.58–0.61
High	0.86–1.32	0.02–0.16	−14.00–16.00	−1.98–13.00	−1.19–10.00	0.70–6.50	−1.44–0.43	−3.31–0.38

## Data Availability

The data that support the findings of this study are available from the corresponding author upon reasonable request.

## Supplementary Materials

The following supporting information can be downloaded at: https://www.mdpi.com/article/10.3390/ani13061025/s1; File S1: The numerical simulation and validation results.
