# Supplementary material for: Differences in the Natural Swimming Behavior of Schizothorax prenanti Individual and Schooling in Spatially Heterogeneous Turbulent Flows
Source: Animals (Basel). 2023 Mar 10;13(6):1025. doi: 10.3390/ani13061025 (PMC10044503; doi:10.3390/ani13061025)
Supplement: Supplementary file 1 [file animals-13-01025-s001.zip › animals-2177048-supplementary File S1.pdf]

The numerical simulation and validation results

A computational fluid dynamics (CFD) software Flow-3D was used to simulate the fine flow field in the flume to obtain the additional hydraulic variable of the strain rate (SR). The SR reflects the deformation degree of turbulent flow and is defined as:

$$SR_{ij} = \left(\frac{\partial u_i}{\partial x_j} + \frac{\partial u_j}{\partial x_i}\right)/2 \quad (1)$$

The renormalization group k-ε turbulence model (RNG k-ε) was selected to solve the three-dimensional Navier-Stokes equation. Structural hexahedron grids were adopted to geometrically express the entire calculation area with 1.52 million grids in total. Based on the experimental data, both inlet flow rate (0.60 m^3/s) and water elevation (0.41 m) were imposed as the inlet boundary conditions, and the outlet water level (0.39 m) was employed as the outlet boundary condition. Specific pressure boundary was applied for the top of the domain and the regular wall boundary condition was used for all walls. Converged solutions were achieved by running for 800 s with time step of 0.01 s. The flow velocity obtained from the numerical simulation were compared with the measured results to validate the numerical model and assess the accuracy of the simulation results.

(1) Numerical simulation results

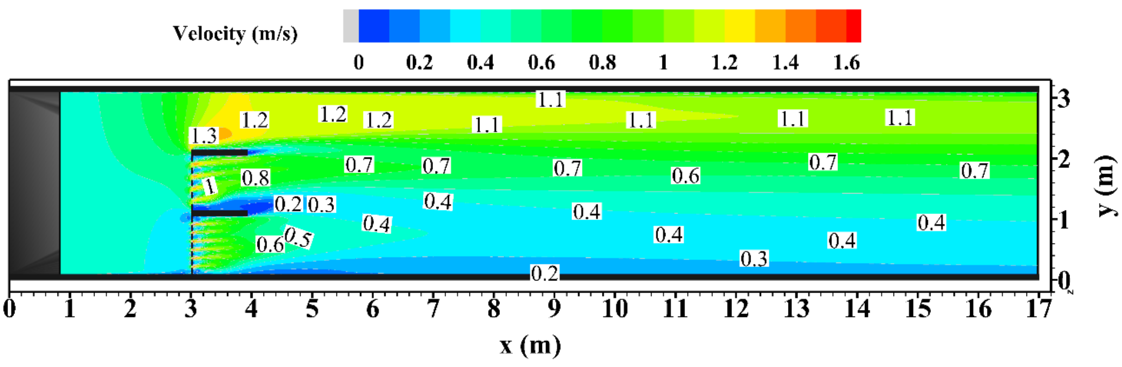

Figure S1 Contour plot of flow velocity (m/s).

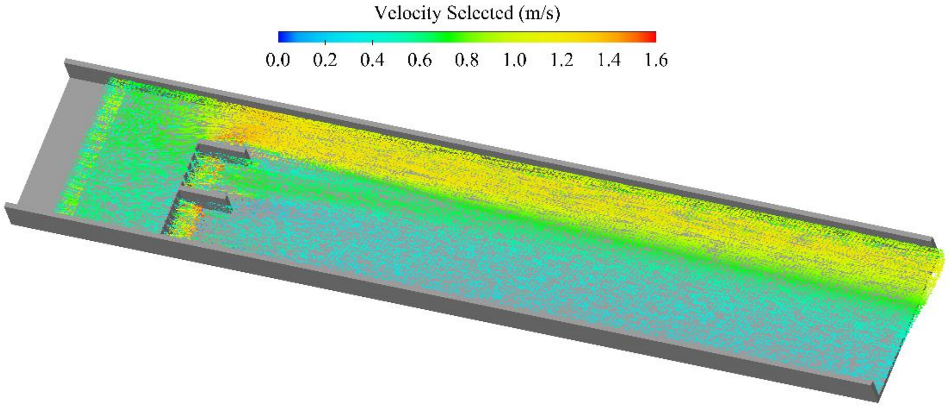

Figure S2 Three-dimensional velocity vector.

(2) Validation result

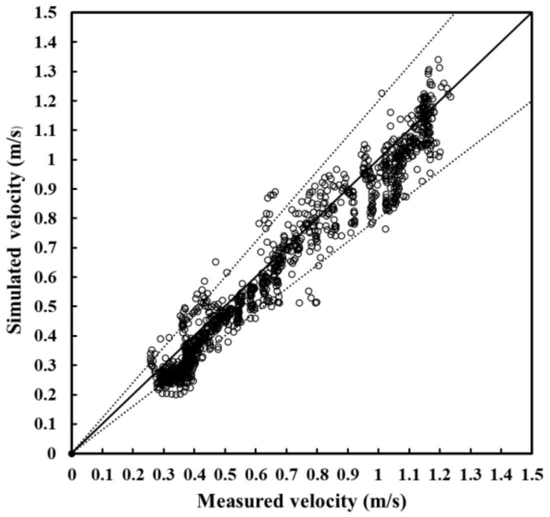

Figure S3 Comparison of the measurement and simulation flow velocity (m/s) results. The dashed line represents 20% error.
